# Synaptic Plasticity in the Pain-Related Cingulate and Insular Cortex

**DOI:** 10.3390/biomedicines10112745

**Published:** 2022-10-28

**Authors:** Jung-Hyun Alex Lee, Qiyu Chen, Min Zhuo

**Affiliations:** 1 Department of Physiology, Faculty of Medicine, University of Toronto, Medical Science Building, 1 King’s College Circle, Toronto, ON M5S 1A8, Canada; 2 Institute of Brain Research, Qingdao International Academician Park, Qingdao 266199, China; 3 Center for Neuron and Disease, Frontier Institute of Science and Technology, Xi’an Jiaotong University, Xi’an 710049, China; 4 Oujiang Laboratory, Zhejiang Lab for Regenerative Medicine, Vision and Brain Health, Wenzhou 325000, China

**Keywords:** chronic pain, neuropathic pain, anterior cingulate cortex, long-term potentiation, synaptic plasticity

## Abstract

Cumulative animal and human studies have consistently demonstrated that two major cortical regions in the brain, namely the anterior cingulate cortex (ACC) and insular cortex (IC), play critical roles in pain perception and chronic pain. Neuronal synapses in these cortical regions of adult animals are highly plastic and can undergo long-term potentiation (LTP), a phenomenon that is also reported in brain areas for learning and memory (such as the hippocampus). Genetic and pharmacological studies show that inhibiting such cortical LTP can help to reduce behavioral sensitization caused by injury as well as injury-induced emotional changes. In this review, we will summarize recent progress related to synaptic mechanisms for different forms of cortical LTP and their possible contribution to behavioral pain and emotional changes.

## 1. Introduction

It is widely believed now that adult synapses are highly plastic. This type of plasticity is highly robust and may persist for a lifetime, especially for negative memories associated with fear or painful events. Among several possible proposed memory mechanisms, synaptic plasticity in the form of LTP is likely a key cellular mechanism for memory. It is believed that synaptic changes in efficacy play important roles in the formation and expression of fear. The hippocampus, amygdala, and related cortical areas are important for such fear memory [1,2]. In a traditional view, cortical areas that are involved in pain perception are not thought to be highly plastic. Some previous researchers believe that painful information may be ‘diffusely’ distributed in the cortex, while spinal cord synapses play important roles in the coding of the intensity of pain. However, recent studies using both in vivo and vitro approaches clearly indicate that excitatory synapses in these pain perception coding areas are highly plastic. Peripheral injury triggers long-lasting plastic changes in cortical synapses including signaling proteins, postsynaptic receptors, presynaptic vesicle releases, as well as possible structural changes. In this review, we will review the recent progress made in these areas.

## 2. Animal Studies of ACC

### 2.1. Lesions in ACC Reduce Chronic Pain

Lesions to the ACC in animal chronic pain models have been shown to produce antinociceptive or analgesic effects [3]. A recent study investigating changes to the intrinsic neuronal properties of layer II/III ACC pyramidal neurons after nerve injury reported increased frequency in spontaneous excitatory postsynaptic currents (sEPSCs) as well as a decreased inter-spike interval (ISI) and refractory periods indicating increased excitability [4]. The targeted destruction of such ACC neurons is thought to attenuate the effects of chronically elevated synaptic activity and possibly pain-related networks in the ACC observed in models of chronic pain [5]. For example, chemogenetic inhibition of specific ACC neuron populations was found to alleviate hyperalgesia induced by Complete Freud’s adjuvant [6]. Other studies have demonstrated that lesions in the ACC can relieve pain-paired aversion [7]. These studies suggest that the ACC is likely primarily involved in the processing of the affective and sensory components of chronic pain. Consistent with these observations, targeted lesions in the ACC had no effect on acute visceral pain behaviors but attenuated allodynia in mice with chronic visceral pain [8].

### 2.2. Electrical Stimulation and Glutamate Microinjection in the ACC Trigger Pain and Fear

Electrical stimulation of the ACC has been shown to enhance pain response as well as trigger fear-associated behaviors. Activation of cortical fibers projecting from the somatosensory cortex I (S1) to the ACC enhance hyperalgesia and direct stimulation of the rACC also enhances the nociceptive response to colorectal distension (CRD) [9]. Further studies have observed that stimulation frequencies between 20 and 100 Hz delivered to the ACC can enhance aversive pain behavior and potentiate spinal excitatory transmission while higher frequency (120 Hz) stimulation was observed to reduce aversive pain in response to mechanical stimuli [10,11]. In addition, high-intensity electrical and chemical stimulation of metabotropic glutamate receptors (mGluRs) within the ACC produces a faciliatory effect on spinal nociception while also contributing to the formation of long-term contextual and auditory fear memory [12,13]. These data strongly implicate the ACC in playing a role in the integration of pathways related to nociception, emotion, and pain memory.

The use of different pharmacological agents has provided additional insight into the role of ACC in pain. ACC microinjection of glutamate, the major excitatory neurotransmitter in the central nervous system (CNS), can result in an increased nociceptive response in mice with visceral hypersensitivity likely due to increased excitatory pain signal transmission. ACC microinjections of NMDA receptor agonists homocysteic acid (DLH) and kynurenic acid resulted in greater pain responses when injected before applying noxious stimuli and could even induce avoidance learning in the absence of any noxious stimuli, respectively [14]. Other studies show that microinjections of glutamatergic receptor antagonists such as D-AP5, Ro 256981, NASPM, CNQX, and DNQX can significantly attenuate hyperalgesia, allodynia, block conditioned place avoidance, and inhibit behavioral sensitization [15,16]. These data strongly suggest that enhanced excitatory synaptic transmission in the ACC can cause allodynia, consistent with evidence that ACC neurons are hyperexcitable during the development of neuropathic pain [17].

## 3. Human Studies of ACC and IC

### 3.1. Imaging Studies

**Figure 1:**
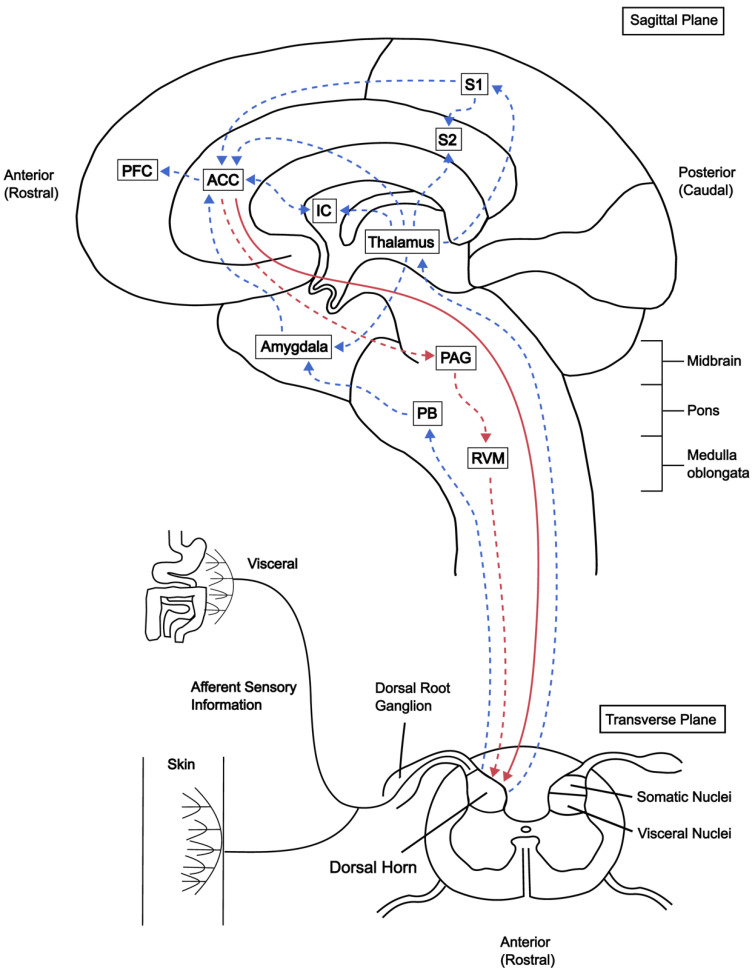
Top–down corticospinal pain and emotion network model. Afferent sensory neurons carry information towards the dorsal root ganglion followed by the somatic and visceral nuclei in the dorsal horn. These sensory neurons project directly to the thalamus but also indirectly to the ACC via the parabrachial nuclei (PB) and amygdala. Conversely, the ACC can modulate spinal dorsal horn neurons both directly or indirectly via the periaqueductal grey (PAG) and rostral ventromedial medulla (RVM). Direct transmission is mediated by glutamate, while indirect pathways involve serotonin (5-HT), which produces facilitatory effects on nociceptive transmission. Artificial stimulation of excitatory ACC neurons can enhance nociception, but activation of inhibitory interneurons produces an analgesic effect suggesting the importance of E/I balance. Beyond this, the ACC also is believed to integrate pain-related sensory and emotional information and relay it to the prefrontal cortex (PFC). Blue arrows indicate ascending projections from the dorsal horn to higher brain structures. Dashed red arrows and the solid red arrow indicate the indirect and direct descending pathway from the ACC to the dorsal horn, respectively. Abbreviations: PFC, prefrontal cortex; ACC, anterior cingulate cortex; IC, insular cortex; S1, somatosensory cortex 1; S1, somatosensory cortex 2; E/I, excitation/inhibition.

Neuroimaging technologies such as positron emission tomography (PET), electroencephalography (EEG), magnetoencephalography (MEG), or functional magnetic resonance imaging (fMRI) can be used to visualize how the brain responds to acute pain, as well as overall changes in different chronic pain states. Acute pain imaging studies of healthy patients show increased activity in cortical structures such as the ACC, IC, S1, and S2 in addition to increased synchronicity in activity across all pain-related cortical structures (cortical pain network) when pairing attention with painful stimuli [18,19]. Structurally, repetitive acute pain stimuli given to healthy patients resulted in increases in gray matter volume in the ACC and prefrontal cortex (PFC) [20]. Thus, spontaneous pain experienced by patients with chronic pain may be explained by frequency-specific overexcitation in pain-related cortical areas. Phantom pain has been previously associated with the reorganization of cortical networks and a new study has provided further evidence that overactivation of cortical areas is present in patients with phantom pain, likely due to changes in functional connectivity [21,22]. In addition, patients with irritable bowel syndrome (IBS) display increased activity in the ACC, IC, and prefrontal cortex (PFC) [23]. Chronic low back pain (CLBP) causes hyperalgesia associated with increased ACC activity and altered functional connectivity, as well as decreased gray matter density in the ACC, PFC, thalamus, brainstem, and somatosensory cortex [24,25]. In cases of fibromyalgia (FM), overall gray matter volume is unchanged but may differ in distribution when comparing healthy to FM patients. A common theme among these studies is increased activity in pain-related cortical areas co-presenting with decreased gray matter density. As the loss of gray matter is associated with the extent of the injury and not necessarily the amount of pain, such as phantom pain, chronic pain may in part be explained by plastic changes at the level of the synapse causing a shift in the excitation/inhibition (E/I) balance of the brain. Thus, changes to the functional connectivity of pain-related cortical networks may lead to overall overexcitation in these networks, despite the loss of neurons in the same areas. The connectivity between the ACC and other brain regions as well as descending projections to the spinal cord establish a top–down corticospinal network involved in pain modulation and the integration of emotion with painful experience (Figure 1).

### 3.2. Electrophysiological Recordings from ACC

Early human electrophysiological studies reported that individual ACC neurons preferentially show increases in firing rate in response to either acute noxious cold stimuli, noxious thermal and mechanical stimuli, or only mechanical stimuli [26]. ACC neurons display differential or graded responses in firing patterns in response to words with high emotional valence and are likely implicated in the salience detection of painful or emotional stimuli [27]. Furthermore, patients with neuropathic pain (chronic pain characterized by shooting or burning sensations) have been shown to display enhanced.

EEG activation within high θ (6–9 Hz) and low β frequencies (12–16 Hz) localized in the IC, ACC, S1, and S2 regions [28]. Suppression of enhanced activity in the ACC in patients with chronic pain and phantom pain using deep brain stimulation (DBS) at frequencies up to 20 Hz resulted in pain relief [29]. Furthermore, the extent of ACC suppression decreased over a 12-month period but was associated with an increase in pain relief. These results may suggest that the suppression of ACC hyperactivity could induce long-term plastic changes in pain-related networks, rescuing changes to the E/I balance in chronic pain states. A recent study combining EEG and fMRI reported increased resting-state brain activity in the form of elevated beta-3 frequency signaling and increased functional connectivity in the ACC in older human patients with elevated pain perception when compared to younger individuals [30].

### 3.3. The IC

The IC is an integrating forebrain structure involved in sensory perception, learning, and memory [31]. It receives afferent projections from thalamic nuclei and forms reciprocal connections with the amygdala, ACC, and other cortical association areas [32,33]. The IC also displays increased activity in response to different noxious stimuli such as gustatory, visceral, mechanical, and somatosensory nociceptive stimuli [34]. Consistent with these data, increasing inhibitory transmission in the IC can produce an analgesic effect and growing evidence strongly suggests that, like the ACC, the IC is indeed a highly plastic region that plays a key role in the psychosocial components of neuropathic pain [35]. A recent study has provided further evidence that the IC is critical for the perception, modification, and chronification of pain and that changes in the functional connectivity of IC networks in clinical studies are associated with changes in white and gray matter in the brain [36,37,38].

## 4. Synaptic Transmission in the ACC and IC

### Excitatory vs. Inhibitory Transmission

**Figure 2:**
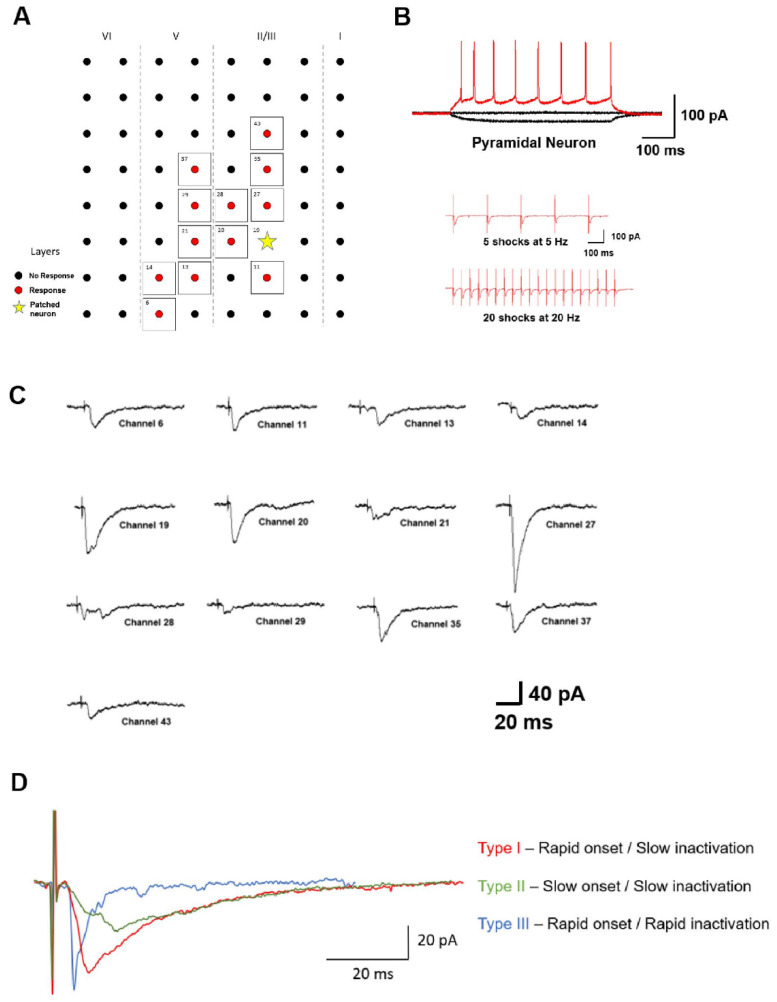
ACC pyramidal neurons receive heterogeneous inputs: (**A**) A representative mapping of a neuronal network within the ACC. The yellow star represents the patched neuron, while the red dots represent channels that elicit a response in the patched neuron when stimulated. Representative traces (right) of the responses recorded in the patched neuron when stimulations were given to different channels. (**B**) Identification of pyramidal type by injecting step currents (−50, 0, and 50 pA). Monosynaptic connectivity was tested using 5 shocks at 5 Hz and 20 shocks at 20 Hz. Responses triggered without failure in the presence of picrotoxin (100 µM) indicate monosynaptic connectivity. (**C**) Sample traces from the recorded neuron show responses with different characteristics. (**D**) Three representative traces are depicted to illustrate the three different types of response kinetics that were observed: Type 1—Rapid onset followed by a slow inactivation phase illustrated in red, Type II—Slow onset followed by a slow inactivation phase illustrated in green, and Type III—Rapid onset followed by rapid inactivation illustrated in blue. Reprinted with permission from Ref. [44] 2021, Jung-Hyun Alex Lee.

Glutamate is the major fast excitatory neurotransmitter in the central nervous system, which includes cortical areas such as the ACC and IC [39,40]. Both α-amino-3-hydroxyl-5-methyl-4-isoxazolepropionic acid (AMPA or AMPARs) and kainate (KA or KARs) receptors have been shown to contribute to fast excitatory postsynaptic currents (EPSCs) within ACC pyramidal neurons [41]. Specific receptor-mediated components of evoked EPSCs of can be pharmacologically isolated by applying the AMPAR-specific antagonist GYKI 53,655 or AMPAR, and KAR-specific antagonist 6-cyano-7-nitroquinoxaline-2,3-dione (CNQX). In addition, genetic deletion of the GluR6 and GluR5 subunits of the KAR completely blocks KAR-mediated EPSCs and KA-activated currents indicating these subunits are necessary for proper receptor function [42]. A recent meta-analysis of glutamate levels in chronically ill patients diagnosed with major depressive disorder (MDD) showed evidence for increased levels of glutamate in the ACC, which could lead to chronic overexcitation of iGluRs [43]. In addition, the same areas simultaneously showed decreased amounts of γ-Aminobutyric acid (GABA), the major inhibitory neurotransmitter. Within the ACC, individual excitatory and inhibitory neurons have been shown to receive numerous heterogeneous inputs, and the activation of different combinations of inputs is believed to underly the mechanism behind the neuronal coding of information [44] (Figure 2).

GABA has been found to mediate inhibitory transmission in both the ACC and IC by binding to its receptors, which hyperpolarizes the postsynaptic neuron via the influx of negatively charged ions [45,46]. The two classes of GABA receptors are GABA_A_ (ligand-gated) and GABA_B_ (metabotropic/G-protein-coupled) receptors, and although both are expressed in the ACC and IC, the transmission of inhibitory postsynaptic currents (IPSCs) is mainly mediated by GABA_A_ receptors in the ACC. This was shown when bath application of picrotoxin (PTX), a specific antagonist of GABA_A_ receptors but not GABA_B_ receptors, completely blocked both spontaneous and evoked IPSCs [47]. This is likely because, in the adult brain, GABA primarily acts through the activation of fast hyperpolarizing GABA_A_ receptors. Although there are few studies regarding the function of GABA_B_ receptors in the ACC, a recent study has shown that blocking ACC GABA_B_ receptors with the GABA_B_ antagonist CGP35348 in a rat model of neuropathic pain induced mechanical hypersensitivity, which was attenuated with the GABA_B_ agonist baclofen [48].

## 5. LTPs: Pre-LTP and Post-LTP

**Figure 3:**
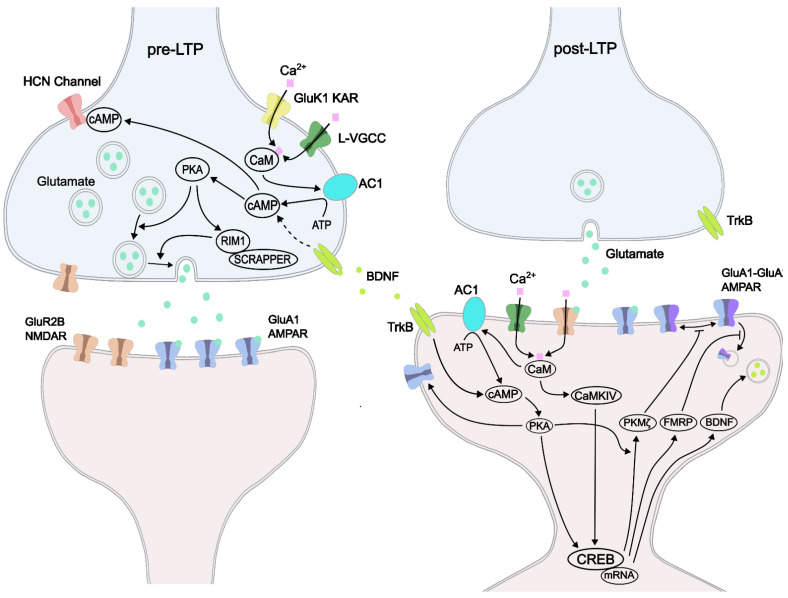
ACC pre-LTP and post-LTP. Two forms of LTP (pre and post) can be observed in the ACC. Presynaptic enhancement of vesicle formation, pool size, and neurotransmitter release probability lead to more readily available agonists of ionotropic glutamate receptors in the synaptic cleft. This can quickly saturate postsynaptic receptors leading to increased calcium influx through the postsynaptic membrane. GluK1-containing kainate receptors (KARs) and ligand-voltage-gated calcium channels (L-VGCCs) allow for the influx of Ca^2+^, which activates calmodulin (CaM). Activation of adenylyl cyclase 1 (AC1) by CaM leads to cAMP and protein kinase A (PKA), which enhances vesicle fusion. Regulating synaptic membrane exocytosis protein 1 (RIM1) together with SCRAPPER, a synapse-localized E3 ubiquitin ligase can upregulate synaptic vesicle release. In addition, activity of the HCN channel with cAMP is believed to modulate the spontaneous release of neurotransmitters. Post-LTP requires the activation of postsynaptic NMDARs, which through the influx of Ca^2+^ can activate the CaMKIV, which activates the cAMP response element-binding protein (CREB). Once activated by cAMP, PKA upregulates the insertion of Ca^2+^ permeable AMPARs to the membrane surface and phosphorylates CREB leading to increased protein synthesis of PKMζ, fragile x mental retardation protein (FMRP), and brain-derived neurotrophic factor (BDNF). BDNF interacts with presynaptic and postsynaptic TrkB receptors and contributes to LTP via the AC1-PKA pathway. De novo synthesis of proteins contributes to increased survival, synaptic plasticity, and neuronal growth, which can persist over a long period of time. As with the importance of PKA, activation of AC1 is also critical for the maintenance of post-LTP.

Pre-existing synapses can undergo activity-dependent modification resulting in either a persisting increase or decrease in synaptic transmission called long-term potentiation (LTP) or long-term depression (LTD), respectively. In the ACC, both presynaptic enhancement of neurotransmitter release (pre-LTP) and postsynaptic receptor upregulation and structural modifications (post-LTP) contribute to synaptic plasticity [49] (Figure 3). In the ACC, persisting enhancement of synaptic transmission can be studied in pyramidal neurons via electrophysiological, pharmacological, and genetic approaches. For example, LTP can be induced using different electrical stimulation protocols such as the pairing training protocol, spike-excitatory postsynaptic potential (EPSP) pairing protocol, and theta-burst stimulation (TBS) [50]. LTP can also be chemically induced in acute brain slices following the washout of 1 mM glycine, which leads to increases in protein kinase M ζ (PKMζ) levels also observed in neuropathic mouse pain models [51]. In addition, LTP can also be chemically induced through a bath application of calcitonin gene-related peptide (CGRP) or brain-derived neurotrophic factor (BDNF) [52,53,54].

Within the ACC, the activation of postsynaptic ligand-gated voltage-dependent N-methyl-D-aspartate (NMDA or NMDARs) receptors are the main driver of postsynaptic LTP but not L-type calcium channels (L-VGCCs) [55]. While not necessary for the induction of post-LTP, L-VGCCs likely play an important role in the induction of pre-LTP through presynaptic modifications such as an increase in the release probability of neurotransmitter-containing vesicles or an increase in vesicle release sites [56]. In ACC pyramidal cells, NMDARs containing GluN2A or GluN2B subunits contribute most to NMDAR-mediated EPSCs, which are almost completely abolished after bath application of both GluN2A antagonist NVP-AAM077 and GluN2B antagonist ifenprodil/Ro compounds. In contrast, blocking either the GluN2A or GluN2B subunits, but not both, only resulted in a reduction in total LTP. Preventing Ca^2+^ interactions via postsynaptic injection of the extracellular Ca^2+^ chelator BAPTA can completely block the induction of LTP. Adenylyl cyclase types 1 (AC1) and 8 (AC8) are highly expressed in ACC neurons and are regulated by Ca^2+^ via CaM [57]. The genetic deletion of AC1 and AC8 can eliminate behavioral sensitization, block the formation of fear memory, and significantly reduce chronic pain [58,59,60]. Furthermore, post-LTP is abolished in AC1 knockout mice suggesting that CaM is critical for the induction of LTP in the ACC. AC1 generates cyclic AMP, which activates protein kinase A (PKA), and this is required for LTP transduction in the ACC. A recent study has shown that AC1 is also necessary for the induction of pre-LTP in the IC and that the effects of genetically knocking out AC1 could be mimicked using the selective AC1 inhibitor NB001 [61]. Other signaling proteins or kinases are also involved in the induction or maintenance of ACC LTP such as CaM kinase IV (CaMKIV), early growth response gene 1 (egr1), Rho GTPases, and fragile X mental retardation protein (FMRP) [62,63,64,65,66]. Another CaM-sensitive protein, CaMKIV, is enriched in ACC synapses and is required for the induction of LTP in the ACC where it may function to activate the transcription factor cAMP response element (CRE)-binding protein (CREB). Synaptic plasticity experiments in rat chronic inflammatory models indicate that BDNF can produce ACC LTP which is believed to occur via an interaction with the AC1-PKA signaling pathway leading to activation of CREB [54,62,67].

## 6. LTPs: Early Phase LTP and Late-Phase LTP

Both e-LTP and l-LTP have been reported in ACC neurons from adult mice using spike-timing and TBS stimulation protocols [68]. Early phase LTP (e-LTP) describes the initial component of LTP described primarily by the upregulation of presynaptic activity preceding de novo protein synthesis, which gradually decays in an activity-dependent manner. On the postsynaptic side, the activation of NMDARs facilitates the strong influx of Ca^2+^ into the dendritic spines, which can trigger the activation of calcium/calmodulin-dependent protein kinases (CaMKs), which are involved in ACC neuronal development and synaptic plasticity [69,70]. Activation of CREB via cAMP-activated pathways can lead to structural modifications of synapses through the activity of proteins that upregulate cell proliferation, differentiation, survival, synaptic plasticity, and neurogenesis, all of, which contribute to the formation and persistence of late-phase LTP (l-LTP) [71]. Thus, l-LTP is protein-synthesis-dependent and is characterized by both pre- and postsynaptic modifications. Different phases of LTP were initially described in CA1 fibers of the hippocampus as the proposed mechanism underlying learning and memory by Bliss and Collingridge and the existence of multiple components of synaptic plasticity in the ACC have also been implicated in helping to explain the development and persistence of chronic pain [72,73,74].

Genetic deletion or pharmacological inhibition of adenylyl cyclase 1 (AC1) has been shown to block the induction of both e- and l-LTP highlighting the necessary action of cAMP [75,76]. Under physiological conditions, blocking PKMζ with ζ-pseudosubstrate inhibitory peptide (ZIP) reverses l-LTP indicating a role in the maintenance of l-LTP. When PKMζ is inhibited through ACC microinjections of ZIP in nerve-injured mice, behavioral sensitization is blocked suggesting PKMζ may contribute to neuropathic pain through the maintenance of cortical potentiation. PKA-dependent phosphorylation of GluA1 AMPAR subunits contributes to enhanced synaptic potentiation in mouse models of pain and a recent study has shown that altering the PKA phosphorylation site s845 at GluA1 subunits significantly impaired the expression of l-LTP at both individual synapses and on a network level [76,77]. Nerve injury activates the CREB/BDNF pathway and specific or peripheral inhibition of CREB can reverse hyperalgesia and produce analgesic effects [78,79]. In another study, the application of NASPM, a potent Ca^2+^ permeable (CP) AMPAR antagonist, could reverse LTP several hours after induction suggesting that the postsynaptic recruitment of CP-AMPARs may be necessary for insular late-phase LTP [80,81].

## 7. Long-Term Depression (LTD)

In the ACC, both NMDA-dependent and NMDA-independent forms of LTD have been observed [82]. Stimulation protocols for the induction of LTD are typically lower in frequency and longer in duration compared to LTP induction protocols. For example, pairing presynaptic stimulation with postsynaptic depolarization or using TBS induces NMDAR-dependent LTP in the ACC in whole-cell recordings, whereas low-frequency stimulation (1 Hz) may trigger GluN2B subunit-containing NMDAR-dependent LTD [83,84,85]. The modulation of AMPARs is also involved in the induction but perhaps not the maintenance of LTD, as the application of an exogenous PKC activator led to the internalization of the GluA2 subunit-containing AMPARs leading to LTD [86]. Consistent with this idea, ACC LTD could not be induced in GluA2 knockout mice. However, an isoform of PKC known as PKCλ plays an active role in the induction of LTP via the phosphorylation of GluA1 subunits resulting in the trafficking of AMPARs to active synapses [87]. Blocking GluN2B receptor function using its antagonist, NVP-AAM07 was able to reverse LTD depotentiation highlighting the role of Ca^2+^ conductance via NMDARs [39]. Furthermore, no changes in paired-pulse ratios were observed during ACC LTD suggesting a post-LTD form of synaptic plasticity in the ACC. Unlike ACC LTP, inhibition of either GluN2A or GluN2B subunits is sufficient to block ACC LTD suggesting that both subunits are necessary for ACC LTD induction, and have different roles in ACC LTP and LTD. In an animal model of bone cancer pain, NMDAR-dependent LTD was impaired and Western blot analyses showed a significant reduction in GluN1, GluN2A, and GluN2B subunits [88]. Similarly, a spike-timing-dependent form of ACC LTD was found to be strongly impaired in a chronic constriction injury model, which continued after recovery [89], suggesting that the loss or reduction in LTD persists in chronic pain. A recent study performed multi-channel field recording induction of LTD via long frequency stimulation (LFS) 30 min after triggering TBS LTP and found that this event was able to reverse the potentiation in ACC [90]. In addition, this effect could be blocked using a GluN2B subunit-specific NMDAR antagonist suggesting again a likely role of the GluN2B subunit in NMDAR-dependent forms of LTD.

Another form of LTD which acts primarily through mGluR1s and L-VGCCs has been previously reported in the ACC in a tail amputation mouse pain model [91]. LFS-induced LTD was impaired after injury but could be rescued by co-applying mGluR1 receptor activator (RS)-3,5-dihydroxyphenylglycine and protein kinase C activating MPEP. Bath application of AP5 partially impaired LTD, but the application of either nimodipine (L-VGCC antagonist) or MCPG (mGluR1 antagonist) completely blocked LTD. The impairment of L-VGCCs on presynaptic terminals may inhibit the effects of LFS stimulation by reducing the activity of AC1 as well as neurotransmitter vesicle formation and release. On the postsynaptic terminal, activation of mGluRs can activate signaling cascades which upregulate GluA2 subunit trafficking to the synapse while removing Ca^2+^ permeable AMPARs leading to synaptic depression [92]. Inhibition of these mGluRs likely prevents AMPAR subunit cycling which blocks the induction of LTD. In models of chronic pain, insufficient LTD associated with the dysregulated overactivation of pain-related cortical networks may explain the persistence of pain, and imbalances in the E/I balance could be rescued by upregulating factors involved in LTD such as mGluRs, GABAergic transmission, and L-VGCCs.

## 8. Functional Implications of LTPs and LTDs

Enhanced excitatory synaptic transmission at individual synapses and within pain-related cortical networks may explain the persistence of chronic pain long after injury or recovery. Animal models of chronic pain have shown increases in intrinsic basal synaptic transmission in the ACC, which is consistent with reduced levels of inducible LTP, possibly indicative of synapses that have plateaued in activity. Initially, increased transmission in the ACC in response to pain may primarily be mediated through the activity of presynaptic factors such as SCRAPPER, a ubiquitin ligase that modulates neurotransmitter vesicle release and is necessary for the induction of ACC pre-LTP [93,94]. This early phase of potentiation may eventually lead to the de novo synthesis of both pre- and postsynaptic new proteins resulting in a late-phase LTP [95]. This process has been proposed to be analogous to initial pain states eventually leading to the formation and persistence of chronic pain (late-phase LTP) in parallel with reduced inhibition or LTD. In addition, the formation of new cortical synapses or the recruitment of silent synapses contributing to ACC potentiation in chronic pain models is thought to contribute to cortical disinhibition and descending facilitation [96,97]. Long-lasting synaptic changes which enhance excitatory transmission may effectively cause the brain to remember and relive pain, and the reduction in LTD suggests an alteration in the overall brain E/I balance which has been associated with both neurological and neuropathic conditions [98,99,100,101].

## 9. Synaptic Tagging

Synaptic tagging was first described in the hippocampus as the ability of a synapse to undergo l-LTP in response to a weak stimulation when preceded by a stronger stimulation of another pathway converging onto the same population of neurons [102]. Thus, the Synaptic Tagging and Capture (STC) hypothesis has been used to explain memory storage and allocation [103]. Strong stimulation from one input triggers the production and diffusion of plasticity-related proteins (PRPs) at the target synapse, which can be “captured” and utilized by neighboring synapses when given weak stimulation at a secondary pathway to induce l-LTP [104,105,106]. Additionally, the priming effect of the strong stimulation is inverse to the interval between strong and weak stimulations indicating that synaptic tagging has temporal constraints [107]. Synaptic tagging has been reported in ACC excitatory synapses, and peripheral injury in mice results in the loss of synaptic tagging [108]. A possible explanation is that elevated levels of cortical potentiation as a result of injury results in a state where synapses cannot be potentiated any further, which is consistent with the inability to induce (additional) l-LTP or observe synaptic tagging. In such a case, enhanced synaptic tagging in the ACC may play a role in the development of anxiety-chronic pain interactions and help to explain the persistence of phantom limb pain or post-traumatic stress disorder (PTSD) [109].

## 10. Sex-Related Studies of Cortical Plasticity

Human studies investigating sex differences in pain suggest that different mechanisms of pain transmission and expression of cortical plasticity may occur in males and females [110,111,112,113]. A recent study showed that the transmission of sensitized pain after nerve injury in mice is mediated by microglia in male mice and T cells in females, providing further evidence for sexual dimorphism in pain pathways [114,115,116,117]. Animal studies indicate no differences in l-LTP in ACC or somatosensory cortical neurons observed between healthy male and female mice, although larger levels of LTD were seen in male mice [118,119]. Similarly, no differences in e- or l-LTP are observed in male and female mice at hippocampal SC-CA1 synapses, although male mice show more l-LTP specifically at TA-CA1 synapses [120]. In brain slices of adult female mice, bath application of AC1 inhibitor NB001 blocked the induction of LTP in ACC [121]. In a recent study using an early life chronic mild stress model, only female mice retained enhanced plasticity into adulthood accompanied by increased expression levels of GluN2B (NR2B) subunits in the visual cortex [122]. Other recent studies showed that estradiol (E2) induced LTP is attenuated after the application of PKA inhibitor mPKI or E2-converting AROM inhibitor letrozole in female mice but not male mice suggesting a modulatory effect of E2 on synaptic plasticity [123,124].

## 11. Neuromodulation of ACC Plasticity: Oxytocin, Norepinephrine, 5-HT, and DA

**Figure 4:**
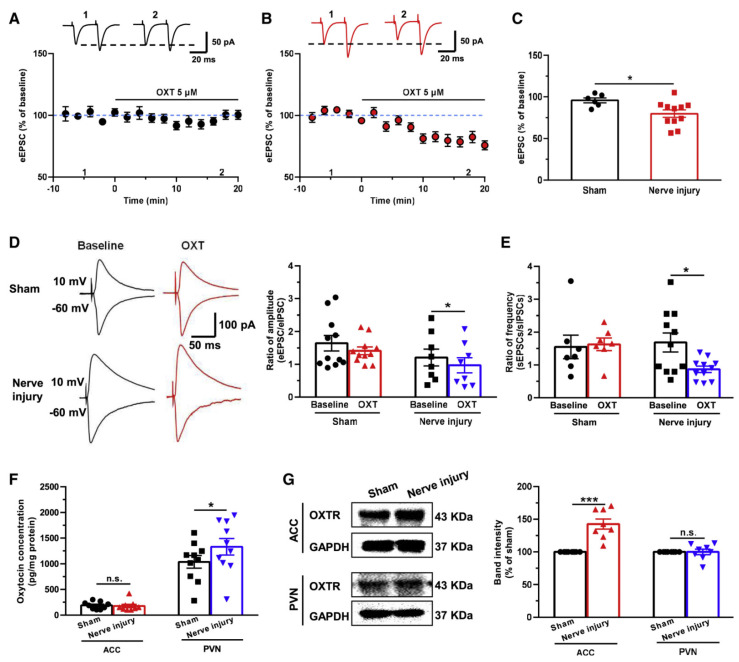
Oxytocin enhances the inhibitory transmission in the ACC after nerve injury. (**A**) Oxytocin did not alter the amplitudes of eEPSCs in the ACC neurons of sham mice (n = 6 neurons/5 mice). (**B**) Oxytocin reduced the amplitudes of eEPSCs in the ACC neurons of nerve-injured mice (n = 11 neurons/5 mice). Representative traces of pair-pulse eEPSCs at baseline (1) and after application of oxytocin (2). (**C**) Summary of the effects of oxytocin on amplitudes of eEPSCs in the ACC neurons of sham and nerve-injured mice. The amplitudes of eEPSCs were statistically different between the sham and nerve injury neurons (* *p* < 0.05, unpaired two-tailed *t*-test). (**D**) Representative traces of evoked excitatory and inhibitory responses were recorded before and after the application of oxytocin in the ACC of sham and nerve-injured mice. The evoked E-I ratio was reduced by application of oxytocin in the nerve-injured mice (n = 8 neurons/6 mice), while unchanged in the sham group (n = 11 neurons/5 mice). Two-way ANOVA, F_(1,34)_ = 4.26, *p* = 0.046. * *p* < 0.05. (**E**) Summary of the effects of oxytocin on the frequency E-I ratio of sEPSCs/sIPSCs in the sham and nerve-injured mice. The frequency E-I ratio of sEPSCs/sIPSCs was reduced by oxytocin in the nerve-injured mice (n = 11 neurons/6 mice), while not changed in the sham group (n = 7 neurons/5 mice). Two-way ANOVA, F(1,32) = 4.67, *p* = 0.032. * *p* < 0.05. (**F**) The oxytocin concentration was tested by ELISA kit in the ACC and PVN after nerve injury (ACC: n = 9 mice/group, *p* > 0.05; PVN: n = 10 mice/group, * *p* < 0.05; unpaired two-tailed *t*-test). n.s. means non-significant. (**G**) Representative Western blots and summary results of oxytocin receptors (OXTRs) expression in total proteins of the ACC and PVN in sham and nerve-injured mice. The oxytocin receptor expression was significantly increased in the ACC after nerve injury compared with the sham group, while not changed in the PVN (ACC: n = 8 mice/group, *** *p* < 0.001; PVN: n = 8 mice/group, *p* > 0.05; unpaired two-tailed *t*-test). n.s. means non-significant. All error bars denote standard errors. Reprinted with permission from Ref. [126]. 2021, Xu-Hui Li.

Interestingly, oxytocin receptor (Oxtr) expression changes over time from mainly excitatory glutamatergic neurons at p14 to GABAergic neurons at p28, where oxytocin perfusion presynaptically enhances inhibitory transmission and reduces excitatory transmission [125]. A recent study combining behavioral and electrophysiological data has indicated that microinjections of oxytocin into the ACC enhance inhibitory transmission via depolarization of inhibitory neurons and reduce chronic-pain-induced anxiety via the reduction in pre-LTP [126] (Figure 4). Additionally, pre-treatment of oxytocin led to decreased ACC activity during early context fear acquisition while post-treatment after nerve ligation attenuated behavioral aversion [127,128]. Oxytocin also alleviated hyperalgesia, as well as inhibited the enhanced expression of GluN2B (NR2B), AC1, PKA, and CREB in a chronic migraine mouse model [129]. Oxytocin is known to modulate fear memory and is also likely involved in fear-pain interactions through projections from the amygdala to the ACC [130]. These results suggest that the increased facilitation of inhibitory transmission via their modulators may have analgesic effects in animal models of pain by balancing out cortical overexcitation.

Norepinephrine/noradrenaline (NE) application produced both pre- and postsynaptic potentiation effects in ACC excitatory transmission in vivo and in vitro [131]. Chronic pain is associated with an impairment to descending noradrenergic modulation and increased anxiodepressive behaviors [132]. Clonidine (NE agonist) has analgesic effects on spontaneous pain and can induce conditioned place preference (CPP) after nerve ligation, which was blocked by inhibiting ACC adrenoreceptors with BRL-44408 maleate [133]. DSP4 (noradrenergic neurotoxin) depletes nearly all ACC noradrenergic neurons without modifying sensory pain perception and increases ERK. On the other hand, desipramine (NE reuptake inhibitor) reduces hyperalgesia and has no effect on ERK levels [134]. Furthermore, 5-HT and NE reuptake inhibitor milnacipran significantly inhibits upregulated ACC activity in response to noxious mechanical stimuli marked by the c-Fos expression [135].

Serotonin (5-HT) bath application in the ACC led to a dose-dependent inhibition of eEPSCs and decreased the frequency of spontaneous and mini EPSCs suggesting inhibitory modulation at both pre and postsynaptic terminals. G protein inhibitor GDP-β-S reversed eEPSC inhibition and 5-HT_1A_ antagonist NAN-190 reversed the 5-HT-induced presynaptic effects [136]. The inhibitory effect of 5-HT was also observed on a network level in a dual whole-cell patch experiment, which showed eEPSCs in the ipsilateral ACC propagated to the contralateral ACC in the presence of a GABA_A_ receptor blockade. Application of ipsilateral 5-HT decreased propagation velocity and increased the contralateral eEPSC latency likely through presynaptic inhibition via 5-HT_1B_ and 5-HT_2A_ receptors or hyperpolarization of ACC excitatory pyramidal neurons through 5-HT_1A_ receptors [137]. In another recent study, activation of ACC 5-HT_7_ receptors reduced enhanced synaptic integration and rescued impaired dendritic hyperpolarization-activated cyclic nucleotide-regulated (HCN) channel function associated with CCI and nerve injury. AC antagonist SQ22526 and HCN antagonist ZD7288 demonstrate that 5-CT activated 5-HT_7_ receptors, which act on HCN channels via activation of adenylate cyclase [138]. Further investigation demonstrated that administration of the novel potent 5-HT_7_ receptor agonist, LP-211, depolarized dendritic membrane potential, accelerated excitatory postsynaptic potential repolarization, and reduced synaptic integration [139]. These data suggest that attenuation of injury-induced cell hyperexcitability via upregulation of 5-HT_7_ receptors may prevent excessive repetitive firing from nociceptive neurons [140], as well as inhibit the strengthening of pain-related cortical networks. Consistent with these data, Nortriptyline (NTP), a selective serotonin–norepinephrine reuptake inhibitor, reduced the injury-induced overactivation of ERK1/2 and relieved chronic behavior, which could be reversed by 5,7-DHT [141].

Dopamine has been shown to modulate the activity of HCN channels within ACC synapses producing an inhibitory effect on pyramidal neurons [142]. Activation of G_s_-coupled D1 receptors causes the opening of HCN channels decreasing the input resistance and membrane excitability of neurons at resting state. In addition, the use of dopamine agonists in neuropathic pain models has been shown to have analgesic effects believed to be the result of the inhibition of hyperexcitable pyramidal neurons via this dopaminergic signaling. Conversely, ACC D1R antagonists blocked the antinociceptive effects of gabapentin and lidocaine suggesting a likely role in chronic pain. These results are in line with evidence associating the dysfunction of dopaminergic neurotransmission with the pain experienced in patients with fibromyalgia or Parkinson’s disease [143]. Furthermore, a study reported a dose-dependent inhibitory effect of dopamine on AMPAR- and KAR-mediated eEPSCs in the ACC, and that this inhibition is driven by postsynaptic D2 receptors [144]. A recent study has reported that D2 receptors are also involved in the regulation of NMDA-dependent LTD in the ACC as downregulation of D2 receptor expression impairs LTD in a self-reporting Drd2 heterozygous SR-Drd^2+/−^ rodent model [145]. For D1 receptors, strong D1R agonists such as SKF 81,297 were found to produce a short-term synaptic depression of AMPAR-mediated currents in the ACC, and the genetic knockout of D1Rs in the ACC enhanced peripheral sensitivity in animal behavior models. These data suggest a role of both D1 and D2 receptors in ACC synaptic transmission related to pain [146].

## 12. Synaptic Structural Changes after Injury

The structural components of synaptic plasticity along with the reorganization of cortical circuits are believed to be the main drivers underlying strong and persistent pain. Described as a form of maladaptive synaptic plasticity, late-stage pain-signaling in cortical regions via increased excitatory and decreased inhibitory synaptic transmission can be explained by long-lasting structural modifications of dendritic spines. A recent study used in vivo structural and functional two-photon imaging to characterize early synaptic abnormalities in a mouse model of Alzheimer’s disease to reveal that the size of dendritic spines was significantly reduced on branches with prolonged calcium transients associated with synaptic depotentiation [147]. The specific structure of individual dendritic spines has been shown to heavily influence calcium dynamics, and changes to these structures in response to peripheral nerve injury may help to explain the observed elevation in transient Ca^2+^ in models of neuropathic pain [148]. In another study, pre-existing spines were shown to have decreased volume and survival following injury whereas newly generated spines postinjury showed a significant and persistent increase in total volume indicative of synaptic remodeling possibly through the action of microglial BDNF [149]. These studies suggest that in both neuropathic pain and disease states, early stages are characterized by a loss or reduction in dendritic spines, which may be followed by an increase in new dendritic spines related to maladaptive signaling and thus cortical reorganization. In vivo calcium imaging of the dorsal ACC revealed that mice with neuropathic pain display hyperactivity of layer V pyramidal neurons characterized by significant increases in both spontaneous and evoked neuronal activity in comparison to sham mice. Furthermore, acute ipsilateral or contralateral peripheral noxious stimuli in control mice produced bilateral responses in ACC layer V neurons [17]. Activity-dependent increases in dendritic spine density paired with a stage-dependent loss of GABAergic terminals may explain the initial onset of neuropathic pain, and further induction of maladaptive synaptic plasticity involving the structural remodeling and reorganization of pain-related cortical networks likely contributes to the continued development and persistence of chronic pain [150].

## 13. Conclusions

Recent evidence strongly points towards a key role of cortical synaptic plasticity in the coding of pain. Upregulation of excitatory synaptic transmission and hyperexcitability of cortical neurons have been repeatedly reported in response to peripheral injury through electrophysiological, pharmacological, and imaging techniques. Parallel to the mechanism believed to underly the coding and long-term storage of learning and memory in the hippocampus, highly plastic cortical neurons may be responsible for the coding and storage of chronic pain. Increased activity in pain-related cortical networks can result in long-lasting potentiation of relevant synapses, which reinforces pain signaling involving additional brain regions such as the amygdala and hippocampus. Thus, the ACC is thought to be a hub that integrates pain information with emotions such as fear and anxiety. Coupled with elevated excitatory transmission and possible changes in neuromodulation, the reduction in LTD and general inhibitory transmission may lead to the disinhibition of cortical networks, disrupting the E/I balance of the brain and further promoting pain signaling. Continued modification of pre- and postsynaptic receptors, involved in the signaling of pain, eventually leads to the production of de novo proteins, which can result in both the formation of new spines as well as changes to the shape and size of dendritic spines. As dendritic branches of neurons are known to compete with one another, the paired loss or reduction in pre-existing dendritic structures before injury effectively causes a rewiring of cortical circuits resulting in unnecessary and persisting pain.

## Data Availability

Further data discussed in this review can be requested by mail to the corresponding author.
